# Supplementary material for: Targeted Mass Spectrometry Reveals Interferon-Dependent Eicosanoid and Fatty Acid Alterations in Chronic Myeloid Leukaemia
Source: Int J Mol Sci. 2023 Oct 24;24(21):15513. doi: 10.3390/ijms242115513 (PMC10649737; doi:10.3390/ijms242115513)
Supplement: Supplementary file 1 [file ijms-24-15513-s001.zip › ijms-2646754-supplementary.pdf]

**Figure S1a:** Optimised eicosanoid and fatty acid profiling by targeted MS. The $MS^2$ ion spectra of $PGF_{2\alpha}$ (molar mass of 354.48) using electrospray ionization in positive mode (ESI+), identifying the sodium adduct ($[M+Na]^+$), potassium adduct ($[M+K]^+$), dimer ($[2M+H]^+$), and the sodiated dimer ($[2M+Na]^+$) adduct of $PGF_{2\alpha}$. The fragments at m/z 319.2 and m/z 265.1, caused by in-source fragmentation, have formulas and structures as shown.

**Figure S1b:** dMRM chromatogram of $PGF_{2\alpha}$. The sodium adduct (precursor ion) is exposed to a CE of 4 eV to obtain the product ion of m/z 360.3, 12 eV for product ion 342.2 m/z, 48 eV for product ion 57 m/z and a 64 eV for product ion 43.3 m/z. The product ion, 360.3 m/z is the most abundant species and is therefore used as the quant ion for determining concentration.

**Figure S1c:** dMRM ion spectra of $PGF_{2\alpha}$. The precursor ion is indicated by the red star at m/z 377.2 The product ions are shown.

**Figure S1d:** Chemical structures of lipids analysed and their proposed product structures. The mass-to-charge (m/z) ratios for the intact structures and product ion structures are shown next to the molecule

**Figure S1e:** Product ion chromatograms of eicosanoid and fatty acid standards. Standards are in an equimolar ratio and an on-column concentration of 300 fmol/L. The chromatogram is not scaled to the largest in the chromatogram, which would make each peak have the same height (normalise the counts to the largest peak in the chromatogram). For each lipid, the most intense peak is the quant ion, the other three peaks are the respective qual ions.

**Figure S2a:** Comparison of liquid-liquid extraction (LLE) methods for cellular lipids. Three LLE techniques were tested. In liquid-liquid Extraction Method 1 cells are lysed with 50% (v/v) MeOH in $H_2O$ (step 1), followed by lipid extraction with MTBE (steps 2 and 3) and finally 50% (v/v) MeOH in $H_2O$ (step 4). In liquid-liquid Extraction Method 2 cells are lysed with 100% MeOH (step 1), followed by lipid extraction with MTBE:$CHCl_3$ (1:1, v/v)(steps 2 and 3), and 50% (v/v) MeOH in $H_2O$ (step 4). In liquid-liquid Extraction Method 3 cell lysis and all lipid extraction steps are performed with 100% ACN. Three biological replicates were used for each technique.

**Figure S2b:** The graph represents the mean of three biological replicates. Light grey bars are the results from Extraction Method 1, dark grey bars are results from Extraction Method 2, and black bars are from Extraction Method 3. Results are normalised. Normalisation parameters: 100% is defined as the largest mean in each data set and the results are given as percentages. For area under the curve (AUC) for each result (not normalised results), see Table S1a.

**Table S1a:** LLE of lipids from cells using three different methods (extractions). BLM was added before extracting to yield a final concentration of 10 pmol/L on column. The mean, SD, standard error of the mean (SEM) and the CV of biological replicates (3 replicates) has been calculated for each lipid in the BLM.

**Table S1b:** LLE of cell supernatant from cells using three different methods (extractions). BLM was added before extracting to yield a final concentration of 10 pmol/L on column. The mean, SD, standard error of the mean (SEM) and the CV of biological replicates (3 replicates) has been calculated for each lipid in the BLM.

**Figure S3a:** Comparison of LLE techniques for secreted lipids in cell supernatant material. Three LLE techniques were tested. In liquid-liquid Extraction Method 1 lipids are first extracted with MeOH:$H_2O$:MBTE (1:1:2, v:v:v), followed by extraction with MTBE (step 2) and 50% (v/v) MeOH in $H_2O$ (step 3). In liquid-liquid Extraction Method 2 lipids are first extracted with MeOH:MTBE:$CHCL_3$ (2:1:1, v:v:v), followed by extraction step with MTBE:$CHCl_3$ (step 2), and 50% (v/v) MeOH in $H_2O$ (step 3). In liquid-liquid Extraction Method 3 all lipid extract steps are performed with 100% ACN. Three biological replicates were used for each technique.

**Figure S3b:** The graph represents the mean of three biological replicates. Light grey bars are the results from Extraction Method 1, dark grey bars are results from Extraction Method 2, and black bars are from Extraction Method 3. Results are normalised. Normalisation parameters: 100% is defined as the largest mean in each data set and the results are given as percentages. For area under the curve (AUC) for each result (not normalised results), see Table S1b.

**Figure S4:** Matrix effect on lipid detection. The workflow followed for determining the matrix effect for each lipid. Three types of samples must be prepared: a pool of all samples (matrix, yellow tube), a standard mixture (the BLM, red tube) and the standard mixture spiked into the matrix (grey tube). First, the intrinsic contribution of the lipid response from the matrix itself (yellow tube) is removed from the standard mixture spiked into the matrix (grey tube) by subtracting the MRM AUC for each metabolite found in the matrix. This calculation gives the BLM response when present in the matrix (orange tube). A matrix RF value is subsequently obtained by dividing the MRM AUC value for the standard mixture (red tube) by the MRM AUC value for the standard mixture when present in the matrix (orange tube). Numeric values for measured matrix RFs are provided in Table S2.

**Table S2:** Calculating the matrix effect: the values used in this experiment.

**Table S3a:** This table shows the concentration in cell samples, for each biological and technical replicate. The concentration units are above each column.

**Table S3b:** Results of cell samples. The mean concentration, SD and the number of samples (N) is used to calculate the p-value. NT samples are compared to IFN$\alpha$ treated samples.

**Table S3c:** Results of cells. Biological replicates: Samples are in biological triplicate. Each biological replicate is run in technical triplicate. From this the mean, SD (std.dev) and the CV % has been calculated for biological replicates.

**Table S3d:** Results of cells. Technical replicates: Each biological replicate is run in technical triplicate. From this the mean, SD (std.dev) and the CV % has been calculated for technical replicates.

**Table S4a:** This table shows the concentration in cell supernatant samples, for each biological and technical replicate. The concentration units are above each column.

**Table S4b:** Results of cell supernatant samples. The mean concentration, SD and the number of samples (N) is used to calculate the p-value.

**Table S4c**: Results of cell supernatants. Biological replicates: Samples are in biological triplicate. Each biological replicate is run in technical triplicate. From this the mean, SD (std.dev) and the CV % has been calculated for biological replicates.

**Table S4d:** Results of cell supernatants. Technical replicates: Each biological replicate is run in technical triplicate. From this the mean, SD (std.dev) and the CV % has been calculated for technical replicates.

**Table S5:** Calibration curves (response curves) are generated using the response of the product ion. On the X axis is the concentration, on the Y axis is the response (AUC). The equation of the linear regression is y=Mx+C, and the accuracy of the trendline is shown by the $R^2$.

**Figure S5:** Response (calibration) curve for $PGF_{2\alpha}$. An increasing concentration of standard (from 100 zmol/L to 50 fmol/L) is injected and the response (AUC), for the quantitation ion increases. The response is plotted against the concentration (in fmol/L).

**Figure S1a**

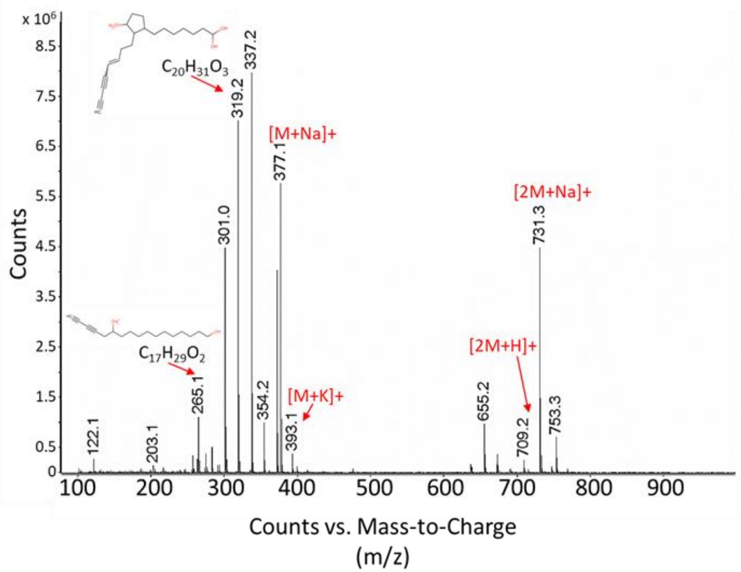


Complete molecular structure of $PGF_{2\alpha}$

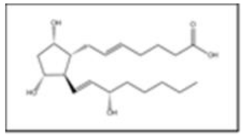

**Figure S1b**

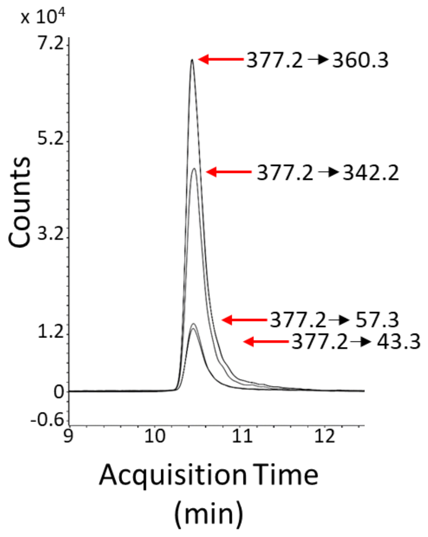
x 10^4
7.2
5.2
3.2
1.2
0
-0.6
Counts
9
10
11
12
Acquisition Time
(min)
377.2 → 360.3
377.2 → 342.2
377.2 → 57.3
377.2 → 43.3

**Figure S1c**

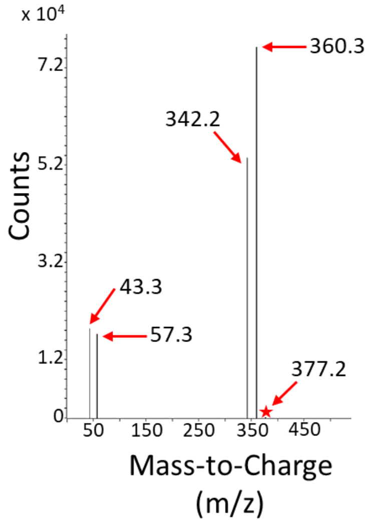
x $10^4$
7.2
5.2
3.2
1.2
0
Counts
360.3
342.2
43.3
57.3
377.2
50
150
250
350
450
Mass-to-Charge
(m/z)

**Figure S1d**

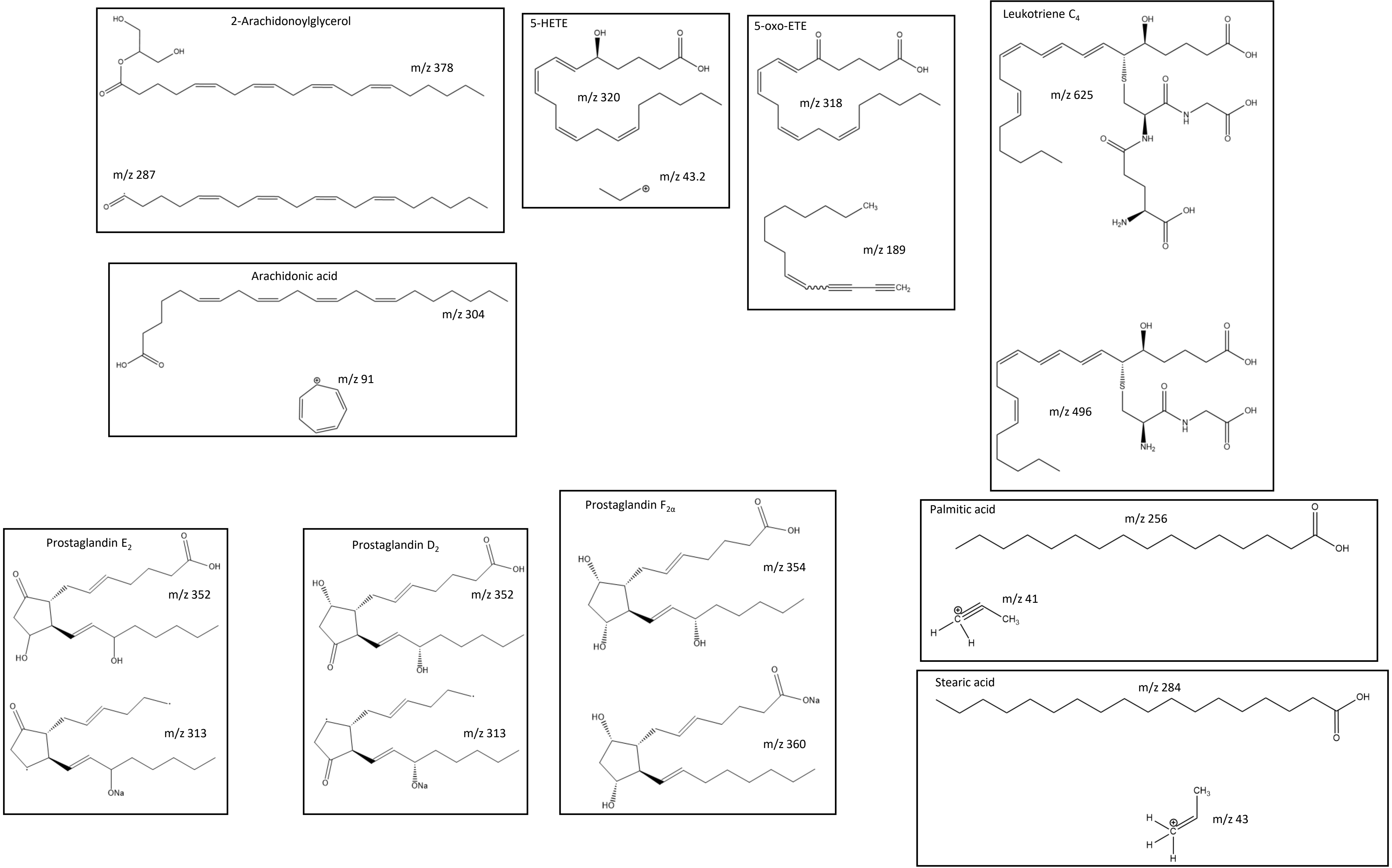
2-Arachidonoylglycerol
m/z 378
m/z 287
5-HETE
m/z 320
m/z 43.2
5-oxo-ETE
m/z 318
m/z 189
Leukotriene C4
m/z 625
m/z 496
Arachidonic acid
m/z 304
m/z 91
Prostaglandin E2
m/z 352
m/z 313
Prostaglandin D2
m/z 352
m/z 313
Prostaglandin F2α
m/z 354
m/z 360
Palmitic acid
m/z 256
m/z 41
Stearic acid
m/z 284
m/z 43

**Figure S1e**

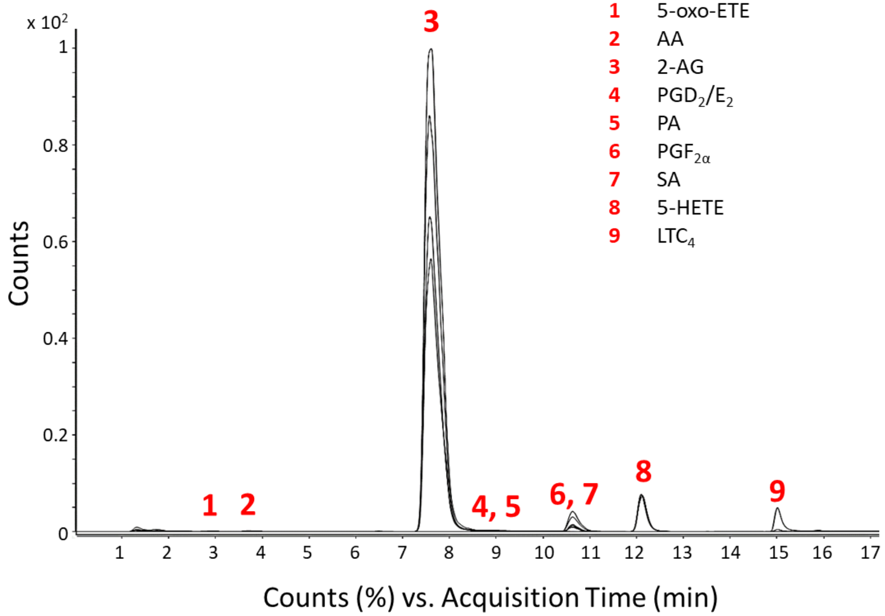


| Lipid | Name |
|---|---|
| 1 | 5-oxo-ETE |
| 2 | AA |
| 3 | 2-AG |
| 4 | $PGD_2/E_2$ |
| 5 | PA |
| 6 | $PGF_{2\alpha}$ |
| 7 | SA |
| 8 | 5-HETE |
| 9 | $LTC_4$ |

Figure S2a

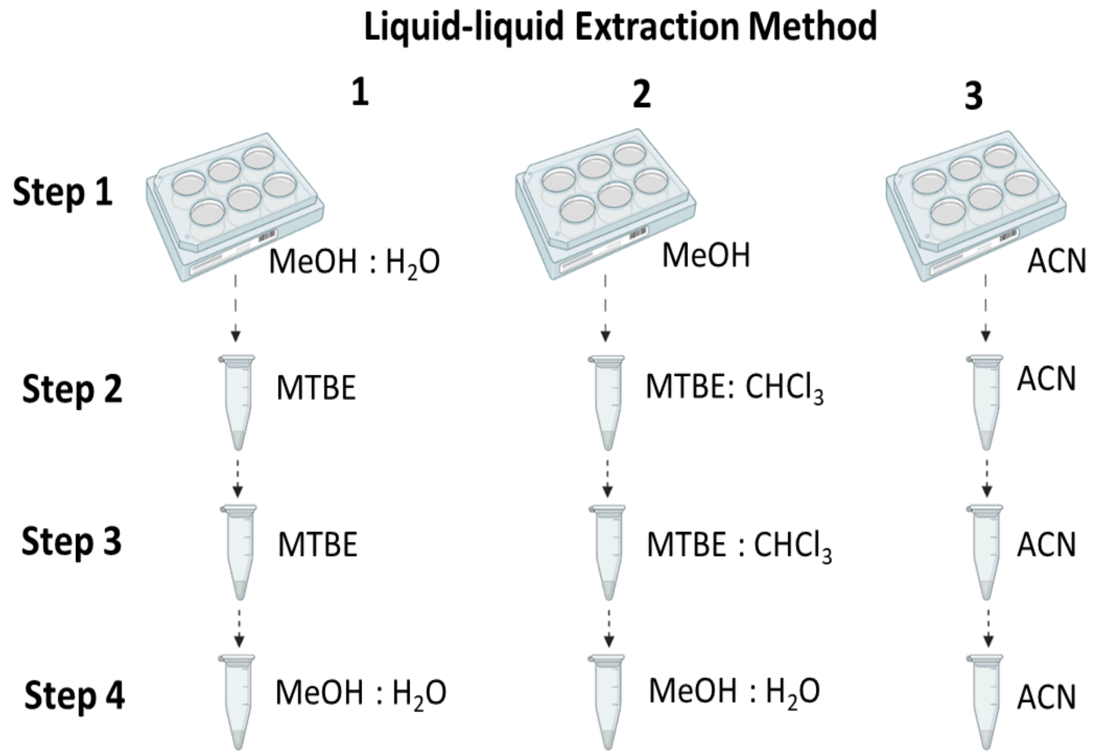

Liquid-liquid Extraction Method
1
2
3
Step 1
MeOH : $H_2O$
MeOH
ACN
Step 2
MTBE
MTBE: $CHCl_3$
ACN
Step 3
MTBE
MTBE : $CHCl_3$
ACN
Step 4
MeOH : $H_2O$
MeOH : $H_2O$
ACN

**Figure S2b**

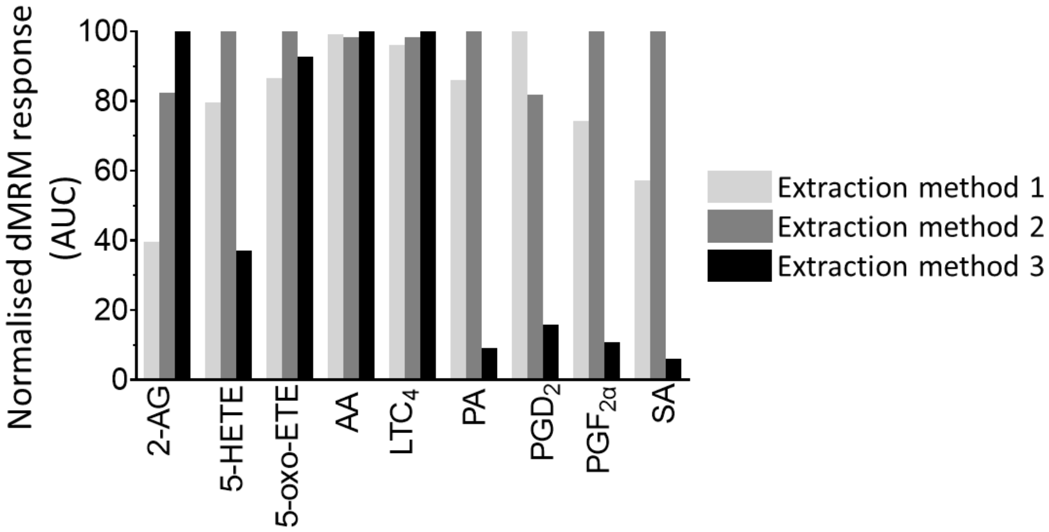
Normalised dMRM response (AUC)
0
20
40
60
80
100
2-AG
5-HETE
5-oxo-ETE
AA
LTC4
PA
PGD2
PGF2α
SA
Extraction method 1
Extraction method 2
Extraction method 3

**Table S1a**

| | Extraction 1 (MeOH/$H_2O$+MTBE) | | | | Extraction 2 (MeOH+MTBE/$CHCl_3$+MeOH/$H_2O$) | | | | Extraction 3 (ACN) | | | |
|---|---|---|---|---|---|---|---|---|---|---|---|---|
| | MEAN | SD | SEM | CV | MEAN | SD | SEM | CV | MEAN | SD | SEM | CV |
| 2-AG | 7.2E+02 | 1.5E+02 | 1.5E+02 | 20.6 | 1.5E+03 | 4.5E+02 | 4.5E+02 | 29.7 | 1.8E+03 | 1.2E+02 | 1.2E+02 | 6.5 |
| 5-HETE | 9.8E+03 | 1.6E+03 | 1.6E+03 | 16.5 | 1.2E+04 | 1.4E+03 | 1.4E+03 | 11.4 | 4.6E+03 | 3.8E+02 | 3.8E+02 | 8.4 |
| 5-Oxo-ETE | 8.3E+03 | 7.4E+02 | 7.4E+02 | 8.9 | 9.6E+03 | 3.2E+02 | 3.2E+02 | 3.3 | 8.9E+03 | 8.3E+02 | 8.3E+02 | 9.4 |
| AA | 1.2E+07 | 1.5E+05 | 3.3E+05 | 1.1 | 1.2E+07 | 1.5E+05 | 1.5E+05 | 1.2 | 1.2E+07 | 8.5E+04 | 8.5E+04 | 0.7 |
| LTC4 | 1.3E+07 | 3.3E+05 | 3.3E+05 | 2.7 | 1.3E+07 | 3.5E+05 | 3.5E+05 | 2.7 | 1.3E+07 | 3.3E+05 | 3.3E+05 | 2.5 |
| PA | 3.5E+04 | 4.0E+03 | 4.0E+03 | 11.5 | 4.0E+04 | 1.1E+04 | 1.1E+04 | 27.8 | 3.7E+03 | 6.1E+02 | 6.1E+02 | 16.4 |
| PGD2/E2 | 1.1E+03 | 5.7E+01 | 5.7E+01 | 5.2 | 8.9E+02 | 2.4E+02 | 2.4E+02 | 27.1 | 1.7E+02 | 7.2E+01 | 7.2E+01 | 41.9 |
| PGF2α | 2.2E+06 | 6.7E+05 | 6.7E+05 | 30.6 | 3.0E+06 | 5.7E+05 | 5.7E+05 | 19.2 | 3.2E+05 | 3.5E+04 | 3.5E+04 | 10.9 |
| SA | 3.8E+05 | 2.7E+05 | 2.7E+05 | 72.8 | 6.6E+05 | 2.4E+05 | 2.4E+05 | 37.2 | 4.0E+04 | 2.3E+03 | 2.3E+03 | 5.7 |

**Table S1b**

| | Extraction 1 ($MeOH/H_2O$+MTBE) | | | | Extraction 2 (MeOH+MTBE/$CHCl_3$+$MeOH/H_2O$) | | | | Extraction 3 (ACN) | | | |
|---|---|---|---|---|---|---|---|---|---|---|---|---|
| | MEAN | SD | SEM | CV | MEAN | SD | SEM | CV | MEAN | SD | SEM | CV |
| 2-AG | 1.0E+03 | 4.4E+02 | 1.5E+02 | 43.7 | 2.1E+02 | 1.8E+02 | 6.1E+01 | 86.9 | 5.0E+02 | 1.3E+02 | 4.5E+01 | 26.9 |
| 5-HETE | 1.1E+03 | 6.9E+02 | 2.3E+02 | 62.0 | 2.9E+03 | 7.7E+02 | 2.6E+02 | 26.2 | 1.8E+03 | 9.0E+02 | 3.0E+02 | 50.4 |
| 5-Oxo-ETE | 6.6E+03 | 7.6E+02 | 2.5E+02 | 11.4 | 1.2E+04 | 2.5E+03 | 8.5E+02 | 20.8 | 8.4E+03 | 5.9E+02 | 2.0E+02 | 7.0 |
| AA | 2.8E+05 | 2.8E+05 | 9.3E+04 | 101.1 | 3.7E+05 | 2.5E+05 | 8.2E+04 | 66.3 | 5.0E+04 | 3.1E+04 | 1.0E+04 | 62.1 |
| LTC4 | 1.4E+07 | 4.2E+06 | 1.4E+06 | 29.6 | 1.3E+07 | 4.3E+06 | 1.4E+06 | 33.4 | 3.8E+06 | 1.0E+05 | 3.4E+04 | 2.7 |
| PA | 2.5E+04 | 1.4E+03 | 4.6E+02 | 5.5 | 2.4E+04 | 2.0E+04 | 6.6E+03 | 83.3 | 3.5E+04 | 8.4E+03 | 2.8E+03 | 23.8 |
| PGD2/E2 | 9.6E+02 | 1.7E+02 | 5.8E+01 | 18.1 | 7.6E+02 | 4.3E+02 | 1.4E+02 | 56.4 | 1.2E+03 | 3.1E+02 | 103.9 | 25.4 |
| PGF2α | 2.6E+06 | 2.0E+05 | 6.6E+04 | 7.5 | 2.2E+06 | 1.3E+06 | 4.3E+05 | 59.0 | 3.2E+06 | 5.5E+05 | 1.8E+05 | 16.9 |
| SA | 3.7E+05 | 3.9E+04 | 1.3E+04 | 10.6 | 3.4E+05 | 2.9E+05 | 9.5E+04 | 83.9 | 4.9E+05 | 1.2E+05 | 3.9E+04 | 24.1 |

**Figure S3a**

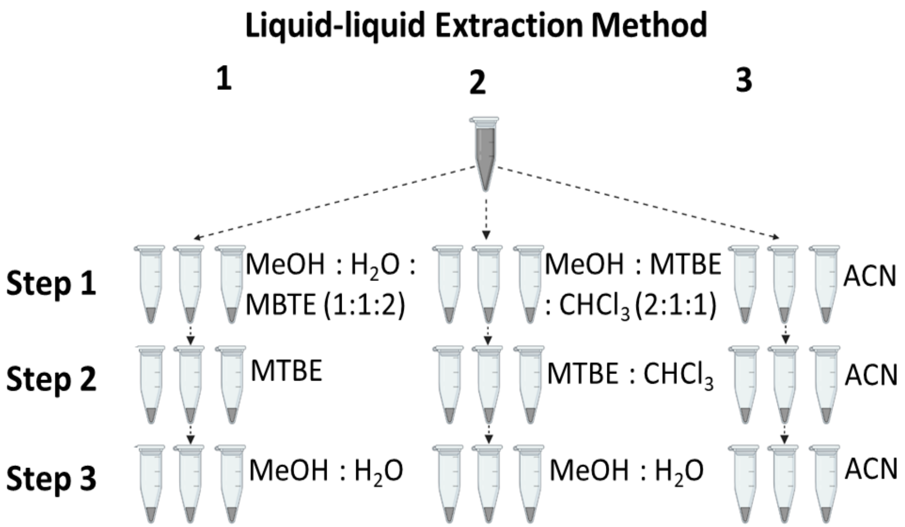

Liquid-liquid Extraction Method
1
2
3
Step 1
Step 2
Step 3
MeOH : $H_2O$ : MBTE (1:1:2)
MeOH : MTBE : $CHCl_3$ (2:1:1)
ACN
MTBE
MTBE : $CHCl_3$
ACN
MeOH : $H_2O$
MeOH : $H_2O$
ACN

**Figure S3b**

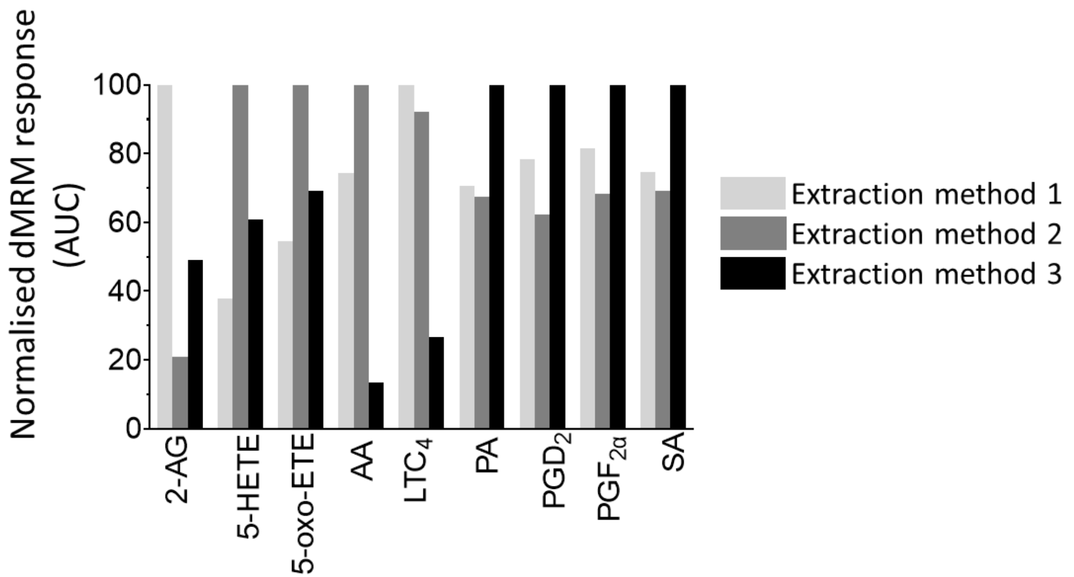
Normalised dMRM response (AUC)
100
80
60
40
20
0
2-AG
5-HETE
5-oxo-ETE
AA
LTC4
PA
PGD2
PGF2α
SA
Extraction method 1
Extraction method 2
Extraction method 3

**Figure S4**

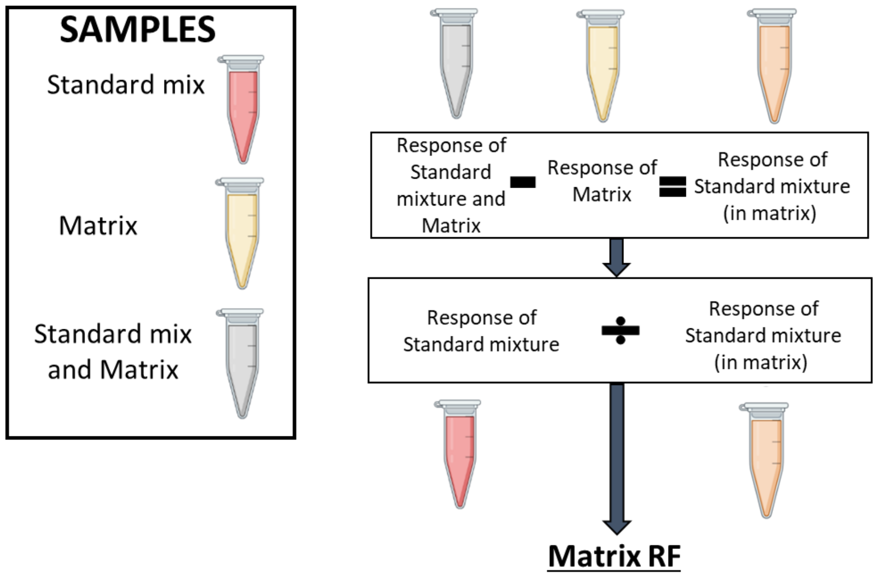
SAMPLES
Standard mix
Matrix
Standard mix and Matrix
Response of Standard mixture and Matrix
Response of Matrix
Response of Standard mixture (in matrix)
Response of Standard mixture
Response of Standard mixture (in matrix)
Matrix RF

**Table S2**

| Matrix RF | AA | 2-AG | PA | $PGF_{2\alpha}$ | SA | 5-HETE | $LTC_4$ |
|---|---|---|---|---|---|---|---|
| **Cells** | 28.97 | 0.34 | 40.06 | 3.95 | 5.51 | 1.11 | 6.17 |
| **Cell supernatants** | 1.25 | 0.93 | 0.07 | 0.09 | 0.11 | 0.58 | 4.81 |

**Table S3a**

| Concentration unit | AA<br>nmol/L | 2-AG<br>pmol/L | PA<br>µmol/L | $PGF_{2\alpha}$<br>pmol/L | SA<br>nmol/L | 5-HETE<br>pmol/L | $LTC_4$<br>µmol/L |
|---|---|---|---|---|---|---|---|
| NT Biological rep 1 Technical rep 1 | 1.23 | 109.19 | 11.59 | 349.62 | 0.71 | 30.70 | 74.65 |
| NT Biological rep 1 Technical rep 2 | 1.29 | 90.18 | 7.31 | 271.27 | 0.44 | 24.47 | 72.31 |
| NT Biological rep 1 Technical rep 3 | 1.40 | 70.92 | 10.79 | 314.60 | 0.55 | 27.13 | 75.09 |
| NT Biological rep 2 Technical rep 1 | 0.93 | 59.46 | | 544.90 | 1.04 | 39.84 | 75.04 |
| NT Biological rep 2 Technical rep 2 | 1.10 | 59.99 | | 403.42 | 0.68 | 29.95 | 76.65 |
| NT Biological rep 2 Technical rep 3 | 1.02 | 68.62 | | 448.05 | 0.84 | 30.32 | 75.37 |
| NT Biological rep 3 Technical rep 1 | 0.89 | 57.50 | 9.96 | 199.06 | 0.19 | 18.62 | 71.97 |
| NT Biological rep 3 Technical rep 2 | 1.00 | 44.20 | 6.80 | 228.00 | 0.25 | 18.71 | 72.36 |
| NT Biological rep 3 Technical rep 3 | 0.90 | 67.47 | 7.03 | 276.50 | 0.31 | 21.93 | 73.64 |
| +IFNα Biological rep 1 Technical rep 1 | 0.96 | 50.65 | 15.43 | 434.26 | 0.58 | 32.53 | 67.80 |
| +IFNα Biological rep 1 Technical rep 2 | 0.99 | 51.45 | 10.67 | 357.88 | 0.36 | 27.19 | 68.80 |
| +IFNα Biological rep 1 Technical rep 3 | 0.80 | 62.60 | 12.00 | 338.66 | 0.34 | 24.46 | 74.41 |
| +IFNα Biological rep 2 Technical rep 1 | 0.91 | 59.73 | 13.26 | 482.49 | 0.65 | 38.07 | 72.26 |
| +IFNα Biological rep 2 Technical rep 2 | 0.84 | 52.03 | 12.65 | 433.06 | 0.52 | 31.11 | 72.10 |
| +IFNα Biological rep 2 Technical rep 3 | 0.81 | 56.56 | 8.35 | 325.62 | 0.33 | 20.33 | 78.70 |
| +IFNα Biological rep 3 Technical rep 1 | 0.84 | 62.32 | 8.76 | 369.96 | 0.46 | 23.36 | 73.20 |
| +IFNα Biological rep 3 Technical rep 2 | 0.82 | 48.96 | 8.77 | 461.09 | 0.58 | 26.19 | 72.64 |
| +IFNα Biological rep 3 Technical rep 3 | 0.81 | 49.60 | 9.78 | 369.87 | 0.38 | 18.86 | 71.42 |

**Table S3b**

| | NT | | | IFNα | | | p-value |
|---|---|---|---|---|---|---|---|
| Metabolite | Mean | SD | N | Mean | SD | N | NT vs. IFNα |
| Arachidonic acid | 1.0844 | 0.1838 | 9 | 0.8644 | 0.0709 | 9 | 0.0041 |
| 2-Arachidonoylglycerol | 69.7256 | 19.3156 | 9 | 54.8778 | 5.5000 | 9 | 0.0414 |
| Palmitic acid | 8.9133 | 2.1150 | 6 | 11.0744 | 2.4242 | 9 | 0.0993 |
| Prostaglandin $F_{2\alpha}$ | 337.2689 | 111.5323 | 9 | 396.9878 | 56.5696 | 9 | 0.1712 |
| Stearic acid | 0.5567 | 0.2863 | 9 | 0.4667 | 0.1203 | 9 | 0.3974 |
| 5-HETE | 26.8522 | 6.7930 | 9 | 26.9000 | 6.1346 | 9 | 0.9877 |
| Leukotriene $C_4$ | 74.1200 | 1.6301 | 9 | 72.3700 | 3.1546 | 9 | 0.1587 |

**Table S3c**

| | AA | 2-AG | Palmitic acid | $PGF_{2\alpha}$ | Stearic acid | 5-HETE | Leukotriene $C_4$ |
|---|---|---|---|---|---|---|---|
| NT mean | 1.09 | 69.72 | 5.94 | 337.27 | 0.56 | 26.85 | 74.12 |
| NT std.dev | 0.16 | 14.63 | 0.98 | 95.98 | 0.24 | 5.57 | 1.24 |
| NT CV (%) | 14.88 | 20.98 | 16.56 | 28.46 | 43.91 | 20.76 | 1.67 |
| +IFNα mean | 0.87 | 54.88 | 11.07 | 396.99 | 0.47 | 26.90 | 72.37 |
| +IFNα std.dev | 0.04 | 1.01 | 1.49 | 15.20 | 0.03 | 2.99 | 1.64 |
| +IFNα CV (%) | 4.41 | 1.85 | 13.45 | 3.83 | 6.34 | 11.11 | 2.27 |

**Table S3d**

| | AA | 2-AG | Palmitic acid | $PGF_{2\alpha}$ | Stearic acid | 5-HETE | Leukotriene $C_4$ |
|---|---|---|---|---|---|---|---|
| NT Biological rep 1 mean | 1.31 | 90.09 | 9.90 | 311.83 | 0.57 | 27.43 | 74.01 |
| NT Biological rep 1 std.dev | 0.07 | 15.62 | 1.86 | 32.04 | 0.11 | 2.55 | 1.22 |
| NT Biological rep 1 CV (%) | 5.46 | 17.34 | 18.78 | 10.28 | 19.28 | 9.30 | 1.65 |
| NT Biological rep 2 mean | 1.01 | 62.69 | | 465.46 | 0.85 | 33.37 | 75.69 |
| NT Biological rep 2 std.dev | 0.07 | 4.20 | | 59.06 | 0.15 | 4.58 | 0.69 |
| NT Biological rep 2 CV (%) | 6.81 | 6.70 | | 12.69 | 17.43 | 13.72 | 0.91 |
| NT Biological rep 3 mean | 0.93 | 56.39 | 7.93 | 234.52 | 0.25 | 19.75 | 72.66 |
| NT Biological rep 3 std.dev | 0.05 | 9.53 | 1.44 | 31.95 | 0.05 | 1.54 | 0.71 |
| NT Biological rep 3 CV (%) | 5.07 | 16.91 | 18.18 | 13.62 | 19.74 | 7.81 | 0.98 |
| +IFNα Biological rep 1 mean | 0.92 | 54.90 | 12.70 | 376.94 | 0.43 | 28.06 | 70.33 |
| +IFNα Biological rep 1 std.dev | 0.08 | 5.46 | 2.00 | 41.29 | 0.11 | 3.35 | 2.91 |
| +IFNα Biological rep 1 CV (%) | 8.92 | 9.94 | 15.77 | 10.95 | 25.27 | 11.93 | 4.14 |
| +IFNα Biological rep 2 mean | 0.85 | 56.11 | 11.42 | 413.72 | 0.50 | 29.84 | 74.35 |
| +IFNα Biological rep 2 std.dev | 0.04 | 3.16 | 2.19 | 65.49 | 0.13 | 7.30 | 3.07 |
| +IFNα Biological rep 2 CV (%) | 4.87 | 5.63 | 19.15 | 15.83 | 26.60 | 24.45 | 4.14 |
| +IFNα Biological rep 3 mean | 0.83 | 53.63 | 9.10 | 400.31 | 0.47 | 22.80 | 72.42 |
| +IFNα Biological rep 3 std.dev | 0.01 | 6.15 | 0.48 | 42.98 | 0.08 | 3.02 | 0.74 |
| +IFNα Biological rep 3 CV (%) | 1.32 | 11.48 | 5.25 | 10.74 | 16.64 | 13.24 | 1.03 |

**Table S4a**

| | AA | 2-AG | PA | $PGF_{2\alpha}$ | SA | 5-HETE | $LTC_4$ |
|---|---|---|---|---|---|---|---|
| Concentration unit | pmol/L | fmol/L | pmol/L | pmol/L | pmol/L | pmol/L | pmol/L |
| NT Biological rep 1 Technical rep 1 | 44.02 | 3.37 | 47.35 | 1.93 | 0.79 | 0.75 | 342.14 |
| NT Biological rep 1 Technical rep 2 | 47.50 | 1.38 | 31.82 | 1.96 | 0.81 | 0.78 | 343.55 |
| NT Biological rep 1 Technical rep 3 | 39.73 | 1.52 | 10.35 | 1.93 | 0.83 | 0.74 | 343.93 |
| NT Biological rep 2 Technical rep 1 | 42.06 | 0.72 | 27.16 | 2.15 | 0.92 | 0.69 | 339.96 |
| NT Biological rep 2 Technical rep 2 | 38.55 | 0.75 | 0.00 | 2.02 | 0.82 | 0.63 | 334.42 |
| NT Biological rep 2 Technical rep 3 | 176.37 | 1.04 | 17.23 | 2.19 | 0.83 | 0.56 | 337.21 |
| NT Biological rep 3 Technical rep 1 | 134.91 | 0.74 | 18.69 | 2.93 | 0.85 | 0.51 | 334.80 |
| NT Biological rep 3 Technical rep 2 | 84.98 | 0.79 | 20.62 | 2.54 | 0.81 | 0.52 | 332.93 |
| NT Biological rep 3 Technical rep 3 | 66.99 | 0.47 | 14.88 | 2.57 | 0.84 | 0.57 | 315.89 |
| +IFNα Biological rep 1 Technical rep 1 | 45.21 | 0.36 | 156.27 | 3.07 | 1.86 | 1.18 | 341.19 |
| +IFNα Biological rep 1 Technical rep 2 | 40.74 | 0.50 | 145.35 | 3.85 | 2.09 | 1.21 | 327.66 |
| +IFNα Biological rep 1 Technical rep 3 | 46.10 | 0.72 | 107.46 | 3.44 | 2.31 | 1.13 | 352.38 |
| +IFNα Biological rep 2 Technical rep 1 | 63.83 | 0.52 | 169.52 | 3.38 | 1.89 | 1.68 | 334.00 |
| +IFNα Biological rep 2 Technical rep 2 | 60.17 | 0.40 | 164.09 | 3.12 | 1.89 | 1.77 | 334.49 |
| +IFNα Biological rep 2 Technical rep 3 | 56.29 | 0.48 | 194.06 | 3.04 | 1.82 | 1.68 | 343.59 |
| +IFNα Biological rep 3 Technical rep 1 | 33.38 | 0.44 | 166.54 | 2.85 | 1.83 | 1.60 | 337.76 |
| +IFNα Biological rep 3 Technical rep 2 | 29.85 | 0.33 | 147.09 | 2.56 | 1.93 | 1.53 | 345.85 |
| +IFNα Biological rep 3 Technical rep 3 | 30.86 | 0.43 | 149.62 | 5.67 | 2.28 | 1.49 | 346.78 |

**Table S4b**

| | NT | | | IFNα | | | p-value |
|---|---|---|---|---|---|---|---|
| Metabolite | Mean | SD | N | Mean | SD | N | NT vs. IFNα |
| Arachidonic acid | 75.0122 | 49.2474 | 9 | 45.1589 | 12.7271 | 9 | 0.0974 |
| 2-Arachidonoylglycerol | 1.1978 | 0.8814 | 9 | 0.4644 | 0.1145 | 9 | 0.0249 |
| Palmitic acid | 20.9000 | 13.5131 | 9 | 155.5556 | 23.4410 | 9 | < 0.0001 |
| Prostaglandin $F_{2\alpha}$ | 2.24667 | 0.3543 | 9 | 3.4422 | 0.9128 | 9 | 0.0021 |
| Stearic acid | 0.8333 | 0.0371 | 9 | 1.9889 | 0.1909 | 9 | < 0.0001 |
| 5-HETE | 0.6389 | 0.1042 | 9 | 1.4744 | 0.2414 | 9 | < 0.0001 |
| Leukotriene $C_4$ | 336.0922 | 8.6054 | 9 | 340.4111 | 7.6640 | 9 | 0.2774 |

**Table S4c**

| | AA | 2-AG | Palmitic acid | $PGF_{2\alpha}$ | Stearic acid | 5-HETE | Leukotriene $C_4$ |
|---|---|---|---|---|---|---|---|
| NT mean | 75.01 | 1.20 | 20.90 | 2.25 | 0.83 | 0.64 | 336.09 |
| NT std.dev | 22.48 | 0.63 | 6.46 | 0.32 | 0.02 | 0.09 | 6.31 |
| NT CV (%) | 29.97 | 53.03 | 30.91 | 14.07 | 2.17 | 14.44 | 1.88 |
| +IFNα mean | 45.16 | 0.47 | 155.55 | 3.44 | 1.99 | 1.47 | 340.41 |
| +IFNα std.dev | 11.76 | 0.05 | 16.16 | 0.21 | 0.09 | 0.22 | 2.49 |
| +IFNα CV (%) | 26.04 | 11.27 | 10.39 | 6.11 | 4.60 | 15.18 | 0.73 |

**Table S4d**

| | AA | 2-AG | Palmitic acid | $PGF_{2\alpha}$ | Stearic acid | 5-HETE | Leukotriene $C_4$ |
|---|---|---|---|---|---|---|---|
| NT Biological rep 1 mean | 43.75 | 2.09 | 29.84 | 1.94 | 0.81 | 0.76 | 343.21 |
| NT Biological rep 1 std.dev | 3.18 | 0.91 | 15.17 | 0.01 | 0.01 | 0.02 | 0.77 |
| NT Biological rep 1 CV (%) | 7.27 | 43.37 | 50.85 | 0.74 | 1.76 | 2.53 | 0.22 |
| NT Biological rep 2 mean | 85.66 | 0.84 | 22.19 | 2.12 | 0.85 | 0.63 | 337.20 |
| NT Biological rep 2 std.dev | 64.16 | 0.14 | 4.96 | 0.07 | 0.05 | 0.05 | 2.26 |
| NT Biological rep 2 CV (%) | 74.90 | 17.11 | 22.36 | 3.37 | 5.53 | 8.13 | 0.67 |
| NT Biological rep 3 mean | 95.63 | 0.67 | 18.07 | 2.68 | 0.83 | 0.53 | 327.87 |
| NT Biological rep 3 std.dev | 28.73 | 0.14 | 2.39 | 0.18 | 0.02 | 0.03 | 8.51 |
| NT Biological rep 3 CV (%) | 30.04 | 21.53 | 13.20 | 6.74 | 1.87 | 4.98 | 2.59 |
| +IFNα Biological rep 1 mean | 44.01 | 0.53 | 136.36 | 3.46 | 2.09 | 1.17 | 340.41 |
| +IFNα Biological rep 1 std.dev | 2.35 | 0.15 | 20.92 | 0.32 | 0.18 | 0.03 | 10.10 |
| +IFNα Biological rep 1 CV (%) | 5.33 | 28.03 | 15.34 | 9.21 | 8.81 | 2.47 | 2.97 |
| +IFNα Biological rep 2 mean | 60.10 | 0.47 | 175.89 | 3.18 | 1.87 | 1.71 | 337.36 |
| +IFNα Biological rep 2 std.dev | 3.08 | 0.05 | 13.04 | 0.14 | 0.03 | 0.04 | 4.41 |
| +IFNα Biological rep 2 CV (%) | 5.12 | 10.85 | 7.41 | 4.55 | 1.86 | 2.44 | 1.31 |
| +IFNα Biological rep 3 mean | 31.36 | 0.40 | 154.42 | 3.70 | 2.01 | 1.54 | 343.47 |
| +IFNα Biological rep 3 std.dev | 1.48 | 0.05 | 8.63 | 1.40 | 0.19 | 0.05 | 4.05 |
| +IFNα Biological rep 3 CV (%) | 4.73 | 12.60 | 5.59 | 37.98 | 9.57 | 2.96 | 1.18 |

**Table S5**

| Lipid | Linear Regression Equation | $R^2$ |
|---|---|---|
| 5-oxo-ETE | Y = 63948*X + 9971 | 0.982 |
| Arachidonic acid | Y = 78582*X + 16322 | 0.997 |
| 2-Arachidonoylglycerol | Y = 20023*X + 7784 | 0.985 |
| Palmitic acid | Y = 19661.2*X + 1388 | 0.950 |
| Prostaglandin $D_2/E_2$ | Y = 405.2*X + 239.9 | 0.953 |
| Prostaglandin $F_{2\alpha}$ | Y = 2214017*X + 1484422 | 0.980 |
| Stearic acid | Y = 707077*X + 207066 | 0.959 |
| 5-HETE | Y = 3817994*X + 477523 | 0.991 |
| Leukotriene $C_4$ | Y = 794330*X + 2171027 | 0.999 |

**Figure S5**

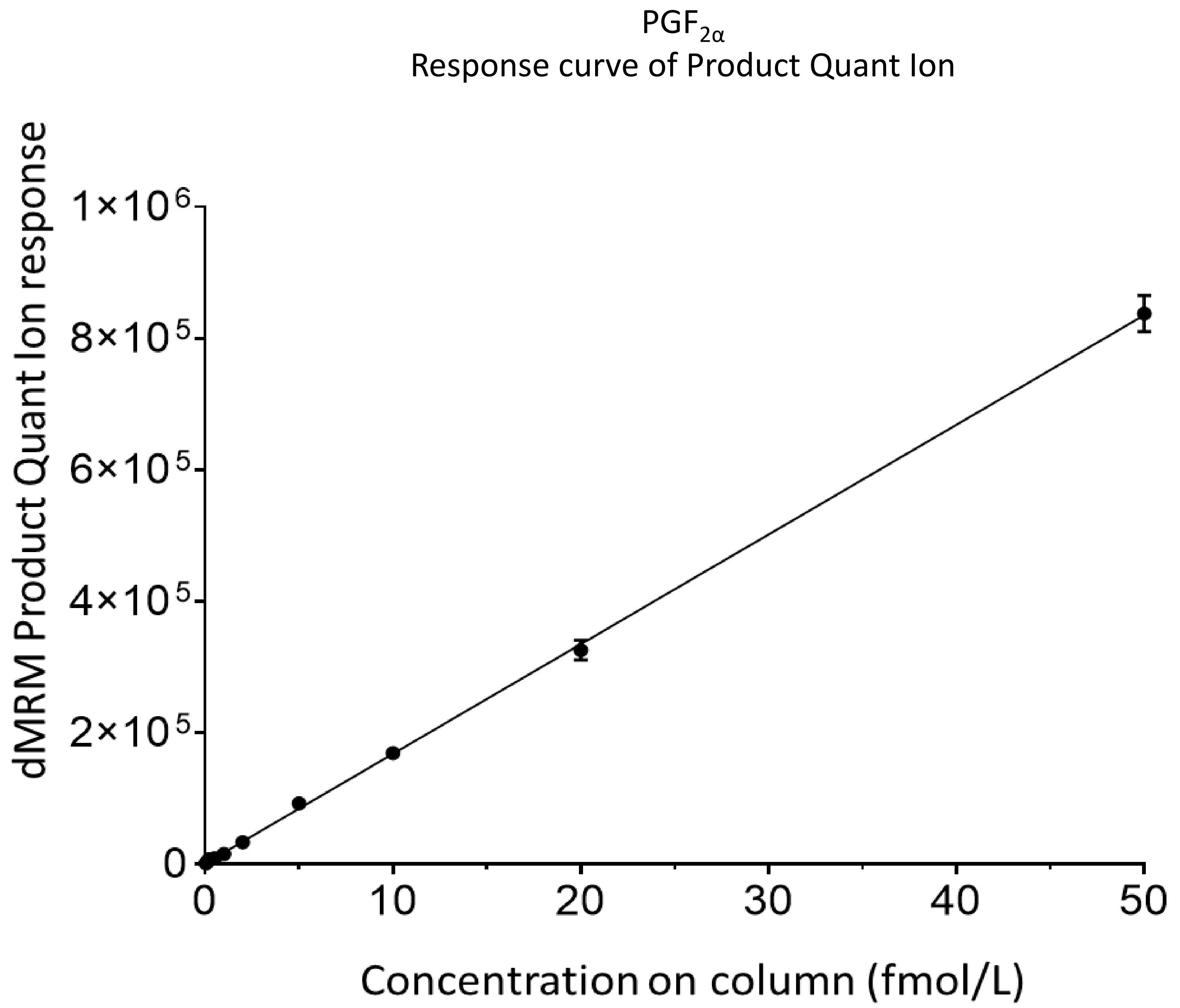
PGF2α
Response curve of Product Quant Ion
dMRM Product Quant Ion response
1×10^6
8×10^5
6×10^5
4×10^5
2×10^5
0
0
10
20
30
40
50
Concentration on column (fmol/L)
